# Comparative Mitogenomic Analysis Reveals Gene and Intron Dynamics in Rubiaceae and Intra-Specific Diversification in *Damnacanthus indicus*

**DOI:** 10.3390/ijms22137237

**Published:** 2021-07-05

**Authors:** Eun-Kyeong Han, Won-Bum Cho, Ichiro Tamaki, In-Su Choi, Jung-Hyun Lee

**Affiliations:** 1 Department of Biological Sciences and Biotechnology, Chonnam National University, Gwangju 61186, Korea; urinara-han@hanmail.net; 2 Department of Plant Variety Protection, Korea Forest Seed and Variety Center, Chungju 27495, Korea; rudis99@korea.kr; 3 Gifu Academy of Forest Science and Culture, 88 Sodai, Mino, Gifu 501-3714, Japan; garageit@gmail.com; 4 School of Life Sciences, Arizona State University, Tempe, AZ 85287, USA; 5 Department of Biology Education, Chonnam National University, Gwangju 61186, Korea

**Keywords:** coffee family, organelle genome evolution, heteroplasmy

## Abstract

The dynamic evolution of mitochondrial gene and intron content has been reported across the angiosperms. However, a reference mitochondrial genome (mitogenome) is not available in Rubiaceae. The phylogenetic utility of mitogenome data at a species level is rarely assessed. Here, we assembled mitogenomes of six *Damnacanthus indicus* (Rubiaceae, Rubioideae) representing two varieties (var. *indicus* and var. *microphyllus*). The gene and intron content of *D. indicus* was compared with mitogenomes from representative angiosperm species and mitochondrial contigs from the other Rubiaceae species. Mitogenome structural rearrangement and sequence divergence in *D. indicus* were analyzed in six individuals. The size of the mitogenome in *D. indicus* varied from 417,661 to 419,435 bp. Comparing the number of intact mitochondrial protein-coding genes in other Gentianales taxa (38), *D. indicus* included 32 genes representing several losses. The intron analysis revealed a shift from *cis* to *trans* splicing of a *nad1* intron (nad1i728) in *D. indicus* and it is a shared character with the other four Rubioideae taxa. Two distinct mitogenome structures (type A and B) were identified. Two-step direct repeat-mediated recombination was proposed to explain structural changes between type A and B mitogenomes. The five individuals from two varieties in *D. indicus* diverged well in the whole mitogenome-level comparison with one exception. Collectively, our study elucidated the mitogenome evolution in Rubiaceae along with *D. indicus* and showed the reliable phylogenetic utility of the whole mitogenome data at a species-level evolution.

## 1. Introduction

The taxonomic perception of intraspecific diversity can provide significant criteria regarding our scientific consensus for ambiguous concepts such as “biodiversity” and “species”. Therefore, infraspecific classification has attracted much attention in studies with a variety of approaches in plant biology (e.g., morphology [1,2], ploidy level [3,4] and phylogeography [5,6]).

The *Damnacanthus* C. F. Gaertn. (Rubiaceae, Rubioideae), an evergreen broad-leaved shrubby genus, consists of about 13 species that are widely distributed throughout tropical to subtropical Asia, including China, Korea and Japan [7,8,9]. The genus represents a unique trait that is heterophylly associated with sympodial growth and paired spines [10,11,12,13]. *Damnacanthus indicus* C. F. Gaertn. exhibits a considerably broad range of leaf variations and is the most widely distributed in the genus [12]. The species comprises a complex of many infraspecific taxa that are primarily endemic to Japan and show a complicated morphological taxonomic structure. For example, in Korea and China, there is no controversy over the taxonomic identity of the species [8,9], while, in Japan, five infraspecific taxa are recorded as being intertwined with *D. major* [7]. They are divided based on the morphological features, such as the size of leaves or the length ratio of leaves and spines. A previous study highlighted that the ploidy level positively correlates with leaf size in the species [12]. However, the infraspecific classification of *D. indicus* has never been tested by a molecular phylogenetic approach.

Plant mitochondrial genomes (mitogenomes) are well known for their highly variable and expanded size, which can be as large as free-living bacteria [14,15]. The hitherto largest known mitogenome (11.7 mega base pairs) was found from *Larix sibirica* (Pinaceae) [16]. However, their size expansion usually does not reflect an increase in mitochondrial (mt) protein-coding genes (PCGs). In fact, the early study of Southern hybridization, in Adams et al. (2002) [17], showed multiple losses of ribosomal (*rpl* or *rps*) and succinate dehydrogenase (*sdh*) genes across the angiosperms. The number of ancestral mt PCGs is estimated as 41 [18]. The 24 (including *matR* gene) PCGs are highly conserved, while 17 of the *rpl*, *rps* and *sdh* genes vary in angiosperms [19]. Adams et al. (2002) [17] showed losses of *rps2*, *rps11*, *rps7*, *rps14*, *rps19*, *rpl2* and *sdh3* genes in Rubiaceae in the three genera (*Coffea*, *Ixora* and *Galium*). The absence of hybridization verifies gene losses. However, the presence of signals can represent intact or pseudogenized genes [20]. As a part of a phylogenomic analysis for the family, Rydin et al. (2017) [21] assembled contigs for mitochondrial regions and identified possible losses of ten genes. The reference mitogenome sequences of Rubiaceae species have not been available until recently (https://www.ncbi.nlm.nih.gov/genome/organelle/; accessed on 30 April 2021).

There are two contrasting evolutionary trends in the plant mitogenome—slow genic evolution and rapid genomic evolution [22]. The slow genic evolution implies a low synonymous substitution rate of functional mt PCGs [23,24]. The rapid genomic evolution corresponds to the recombinogenic nature, which gives rise to drastic genome rearrangement and incorporation of foreign sequences.

The genomic rearrangements in the other organelle genome (i.e., the plastid genome (plastome)) often are regarded as a robust phylogenetic signal [25,26,27,28]. However, these are not utilized for information supporting a phylogenetic clade in mitogenome evolution. In the study of the genus *Monsonia* (Geraniaceae), it was estimated that the infra-generic rearrangement per million years could be high as 35.2, which is not coupled with rates of sequence substitutions [29]. Moreover, studies on intra-specific mitogenome variation demonstrated that a genome rearrangement or a frequency shift between different mitotypes in an individual could occur in a short time [30,31,32]. This extreme genome rearrangement can affect a gene order and shift the splicing mode (from *cis* to *trans*) of introns [33]. In angiosperms, it was estimated that the shift in one of the introns of the *nad1* gene (nad1i728), which contains the *matR* gene within it, occurred at least 15 times [34].

The plant mitogenomes harbor various kinds of sequence elements other than their native DNAs. The most frequently observed exogenous feature is a mitochondrial DNA of plastid origin (MIPT or MTPT) via intracellular gene transfer (IGT) [35,36,37]. Some MIPTs of tRNA genes are known as functional in the mitogenome [38]. However, most MIPTs are not functional in mitochondria and subject to post-IGT substitutions and fragmentations [39].

Mitochondrial sequence data had not been utilized well in angiosperm phylogeny compared to plastid data over several decades [40]. However, in recent years, a growing number of studies showed the potential usefulness of mitochondrial data for phylogenetic analysis [20,21,41,42,43,44,45]. The whole mitogenome can show a level of informative characters equivalent to (or higher than) the whole plastome, due to the overwhelming total length at a species-level taxonomy [43,45].

Because mt PCGs in *Damnacanthus* have never been assessed, we completed and characterized the mitogenomes of *D. indicus*, including var. *indicus* and var. *microphyllus*. To understand the mitogenomic features of the species in a broader evolutionary context, we also acquired and generated mitochondrial data from representative angiosperms and Rubiaceae taxa. Furthermore, the intra-specific variation of the mitogenome structure and sequence divergence were compared for six individuals of *D. indicus* from three natural populations in Korea and Japan. The phylogenomic significance of mitogenomes at a species level is discussed.

## 2. Results

### 2.1. Mitogenome Assemblies and Features

**Figure 1:**
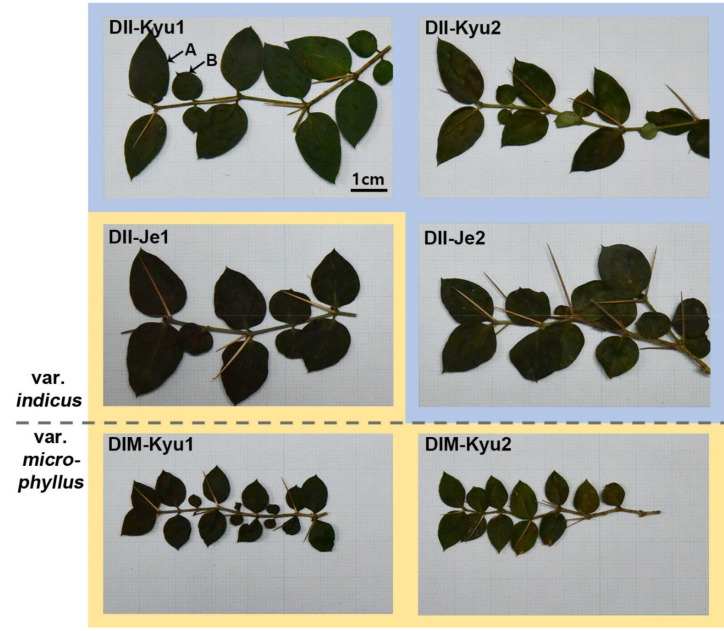
The morphology of six individuals of *Damnacanthus* *indicus*. A dashed horizontal line represents the division between var. *indicus* (upper four) and var. *microphyllus* (lower two). Each individual is marked with its acronym (see Table 1). *D. indicus* is heterophylly with relatively large and small leaves alternately appearing and characterized by a length of spines that is more than half the length of the leaves. The heterophyllous leaves in DII-Kyu1 are indicated (A = large, B = small). *D. indicus* is divided into two varieties according to leaf length; var. *indicus* has a leaf length of 1–2 cm and var. *microphyllus* has a leaf length of 0.5–1 cm. According to our observations, var. *microphyllus* is conspicuously smaller than var. *indicus* in the size of the leaves, the height of the plant and the length of the spines. The colored boxes represent the phylogenetic distinction; the blue part is cluster1 and the yellow network is cluster2. Each image size is equal to 8x5 cm. Scale bar = 1 cm.

**Figure 2:**
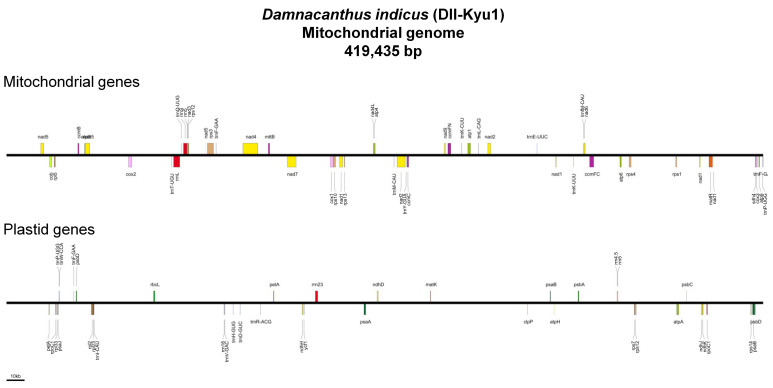
Maps of a representative mitochondrial genome (DII-Kyu1) of *Damnacanthus indicus*. Two maps showing genes of mitochondrial and plastid origins are presented, respectively. Annotations include only intact mitochondrial genes, while for plastid genes, those also include fragmented copies.

The six individuals of *D. indicus* were identified as two varieties (var. *indicus* and var. *microphyllus*) based on morphologies (Figure 1). To distinguish each individual of *D. indicus*, we designated acronyms (e.g., DII-Kyu1 and DII-Kyu2) (Figure 1, Table 1). All mitogenomes were completed as single circular genomes of 417,661–419,435 bp in length (Table 2). Their coverage varied from 66 to 300. Repetitive sequences cover the genomes of 17,550–18,082 bp in length. Mitochondrial gene content was identical among the six individuals and included 3 rRNAs, 12 tRNAs and 32 PCGs (Figure 2). The genomes also include MIPTs of 4 rRNAs, 8 tRNAs and 24 PCGs that are 22,487–22,706 bp in length (Table 2).

### 2.2. Gene Content Comparison with Representative Angiosperms

The content of mitochondrial PCGs of *D. indicus* was compared with several representatives of angiosperms (Table 3). Nine genes are missing (*rpl2*, *rpl10*, *rpl16*, *rps2*, *rps7*, *rps11*, *rps14*, *rps19*, *sdh3*) in *D. indicus*.

### 2.3. Losses of rps7 in Rubiaceae

**Figure 3:**
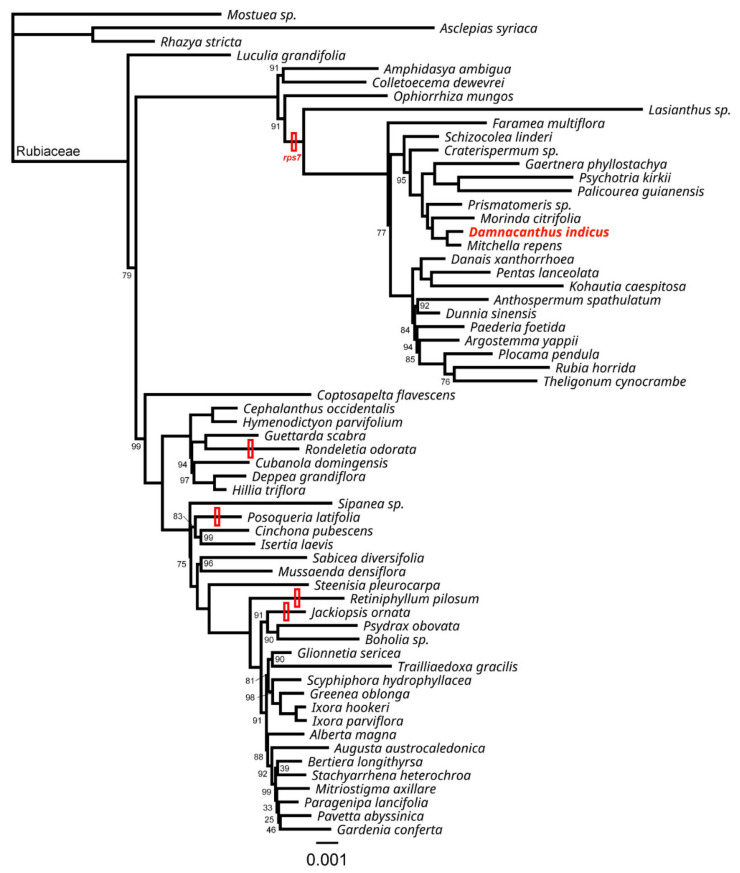
Maximum likelihood phylogeny of Rubiaceae based on concatenated mitochondrial genic regions (40,810 bp in the aligned length). Bootstrap values that are not 100 are shown at the nodes. The scale indicates the number of nucleotide substitutions per site. Branches leading to taxa or taxon with *rps7* loss are marked with empty red bars. The newly sequenced mitogenome of *Damnacanthus indicus* is highlighted with red font.

**Figure 4:**
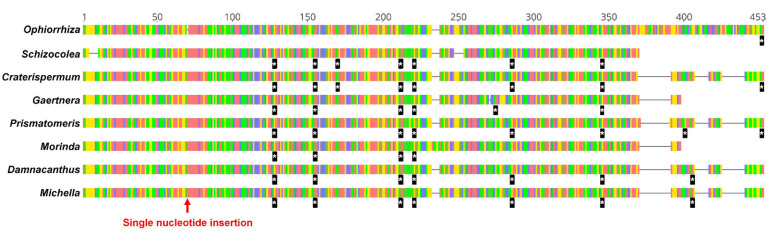
The alignment of *rps7* sequences with a signal of pseudogenization. The sequence of *Ophiorrhiza mungos* is used as a reference. Horizontal lines with four kinds of colors are nucleotides (red = A, green = T, yellow = G, blue = C). Blacklines between nucleotides represent gaps in the alignment. The position of a single nucleotide (A) insertion is marked with a red arrow. The stop codons are presented below each of the nucleotide sequences as a black bar with an asterisk. Omitted species epithets are available in Figure 3.

To understand the gene loss pattern of *rps7*, we reconstructed a phylogenetic tree for the Rubiaceae (Figure 3). There was no *rps7* copy for 18 taxa, while 7 taxa showed pseudogenization among 58 Rubiaceae taxa. Among 25 taxa, 4 (*Rondeletia odorata*, *Posoqueria latifolia*, *Retiniphyllum pilosum* and *Jackiopsis ornata*) showed signals of gene loss of *rps7* and did not form a monophyletic group. However, the rest of the 21 taxa belonged to a highly supported clade (100 bootstrap value), defined by the most recent common ancestor of *Lasianthus sp*. and *Theligonum cynocrambe*. All seven pseudogenized copies of *rps7* were found in this clade. These seven copies showed multiple premature stop codons along with several indels and truncations at 3′ sites, compared to *Ophiorrhiza mungos* (Figure 4). The characteristic shared by all pseudogenized copies was a single nucleotide (A) insertion, making the reading frame shift from the 5′ site.

### 2.4. Trans-Splicing of nad1i728 in Rubiaceae

**Figure 5:**
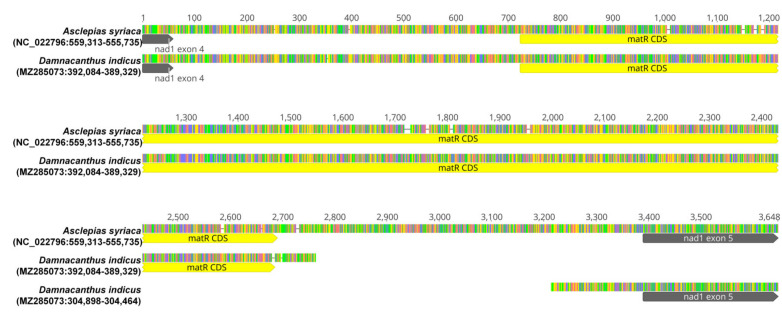
Alignment of the mitochondrial regions of *nad1* exon4, exon5, *matR*, nad1i728 in *Asclepias syriaca* and *Damnacanthus indicus*. Horizontal lines with four kinds of colors are nucleotides (red = A, green = T, yellow = G, blue = C). Black lines between nucleotides represent gaps in the alignment. Below the sequences, the annotations for regions of exons and CDS are presented as dark gray and yellow arrows, respectively. The regions for nad1i728 include *matR* and sequences without arrows.

The intron (nad1i728), which includes the *matR* gene and interferes *nad1* exon 4 and 5, was separated in *D. indicus.* These two separated loci were compared to a locus of *Asclepias syriaca* (Apocynaceae) in Figure 5. The breakpoint of the separation was between the *matR* gene and *nad1* exon5 and showed a 449 bp deletion. The distance between loci for the *nad1* exon4-*matR* region and *nad1* exon 5 was 84,430 bp in DII-Kyu1.

The status of nad1i728 was further surveyed in ten representatives of three subfamilies (Rubioideae, Cinchonoideae and Ixoroideae) of Rubiaceae. It showed that the splicing mode (*trans*) is specific to Rubioideae in the analyzed taxa (Table 4).

### 2.5. Intra-Specific Mitogenome Rearrangement and Divergence of Damnacanthus Indicus

**Figure 6:**
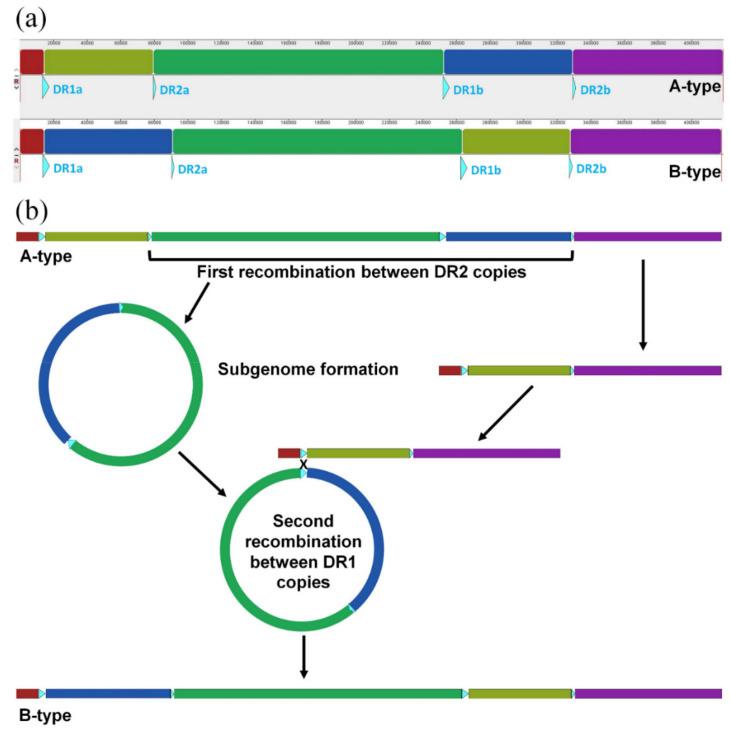
Two alternative mitochondrial genome structures in *Damnacanthus indicus* (**a**) and a plausible molecular scenario of genome rearrangement (**b**). Direct repeats are presented as the sky-blue triangles and their size is larger than their actual size (DR1 = 3500 bp, DR2 = 61 bp). Locally colinear blocks (LCBs) of Mauve alignment are presented as five bars of different colors. The red and purple LCBs are separated in linearized genomes; however, these are a single-connected sequence block in circular form. In scenario (**b**), the A-type is treated as an ancestral type since it is shared by the majority of individuals (five) (Table 5). The first and intramolecular recombination between DR2a and DR2b can generate two smaller subgenomic molecules. Subsequently, the second and intermolecular recombination between DR1a and DR1b can mediate the re-integration of one genomic molecule into the other. Ultimately, the new genome formation (B-type) can be generated.

The Mauve alignment (Figure 6a) showed two different genome arrangements in *D. indicus*. The five individuals showed the same gene order (named A-type), but one individual (DIM-Kyu2) was not colinear to the others (named B-type). The breakpoints of locally colinear blocks (LCBs) coincided with two direct repeat (DR) pairs (DR1 (3000 bp) and DR2 (62 bp)). We provided one of the plausible molecular mechanisms that include the recombination of DR copies (Figure 6b).

Possible structural heteroplasmy was analyzed (Table 5). The analysis confirmed that the two distinct recognized types are major types in each individual. A low-frequency alternative type within three individuals is also shown. In DII-Kyu1 and DII-Kyu2, A-type was a major type and B-type existed as a minor (ca. 1%) type. In DIM-Kyu2, B-type was a major type and A-type was a minor (5%) type.

**Figure 7:**
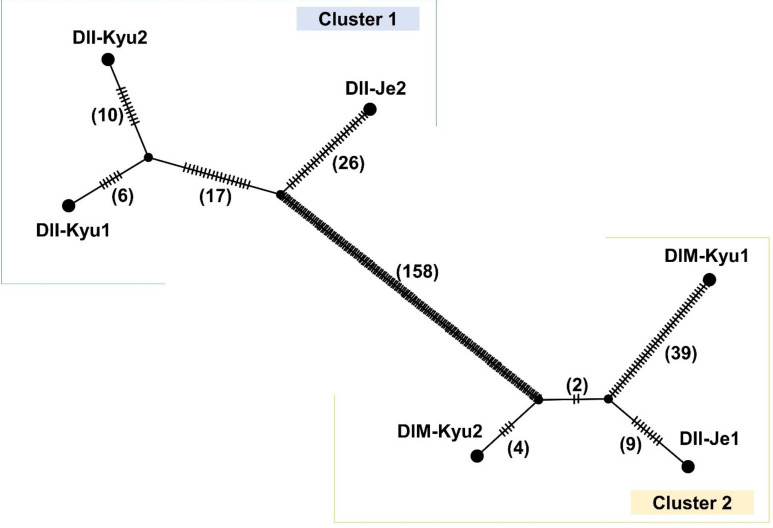
The TCS network based on whole mitochondrial genome sequences of six individuals of *Damnacanthus indicus* (421,524 bp in aligned length). The morphology of these individuals is available in Figure 1. Large black circles at the tips are each of the individual mitochondrial genomes. The small black circles at the nodes are hypothetical missing intermediates. Branch lengths are not directly proportionate to mutational steps. The number of mutational steps is presented as hatch marks and also in brackets. Open boxes represent each cluster.

Intra-specific mitochondrial DNA divergence in *D. indicus* is analyzed in Figure 7. The analysis broadly divided six individuals into two subgroups, diverged by 158 mutational steps. This subgrouping did not support infraspecific classification since two individuals of Jeju Island were divided. DII-Je2 was grouped with other individuals of var. *indicus,* while DII-Je1 was close (15 mutational steps) to DIM-Kyu2 of var. *microphyllus*.

## 3. Discussion

We assembled complete mitogenomes of *D. indicus* as ca. 417–419 kb contigs (Table 2). Their length can be regarded as canonical, since most seed plant species have ca. 200–1000 kb mitogenomes [20]. The genome lengths are smaller than two available Gentianales reference mitogenomes of *Asclepias syriaca* (682,496 bp) and *Rhazya stricta* (548,608 bp) in the Apocynaceae family. Since the mitogenome size in a plant family and genus can be highly variable, it is hard to ascertain whether *D. indicus* represents a mitogenome size reduction of Rubiaceae taxa.

Mitogenomes of *D. indicus* showed a considerable amount of MIPTs covering ca. 23 kb of the genomes containing 36 plastid genes (Figure 2 and Table 2). The extreme entire plastome scale transfer has been reported from an early-diverging angiosperm *Amborella trichopoda* (Amborellaceae) [55]. Many angiosperms have at least a few plastid tRNAs (e.g., *Trifolium* species) [56] and the most common of them is *trnP*-UGG [57], which is also present in *D. indicus*, along with other MIPTs. In *Arabidopsis thaliana* (Brassicaceae), six plastid tRNAs were found in the mitogenome and five of them are regarded as functional in mitochondrion [38,58]. Hence, it is feasible that some of the plastid tRNAs in the *D. indicus* mitogenomes are also functional, but this needs further verification. Apart from tRNAs, most MIPTs are regarded as unfunctional [38,57]. However, these MIPTs can cause false signals in DNA barcoding and NGS sequencing. In NGS sequencing, MIPTs can be bioinformatically sorted out from actual plastid sequences based on their read coverage and sequence divergence [20,59]. However, PCR-based DNA barcoding can co-amplify both inter-compartmental paralogous copies and produce mixed signals in Sanger sequencing [60]. Thus, special attention should be paid to future studies involving analysis on the plastid sequence in the *Damnacanthus* to avoid the inclusion of MIPTs.

Nine genes are missing (*rpl2*, *rpl10*, *rpl16*, *rps2*, *rps7*, *rps11*, *rps14*, *rps19*, *sdh3*) in *D. indicus* from the full 41 PCG set in *Liriodendron tulipifera* (Table 3). The lack of *rps2* and *rps11* genes is the common characteristic of all analyzed taxa (excluding *L. tulipifera*), suggesting that the gene losses occurred earlier than the diversification of Rubiaceae. The other seven gene losses appear to have occurred during Rubiaceae diversification. Previously, Rydin et al. (2017) [21] argued putative gene losses of ten genes (*atp9*, *nad9*, *rpl2*, *rpl10*, *rps1*, *rps7*, *rps10*, *rps19*, *sdh3* and *sdh4*) in Rubiaceae based on reference-based mitochondrial contig assemblies. Thus, the additional loss of five genes *(atp9, nad9, rps1, rps10* and *sdh4)* may occur in other Rubiaceae taxa, even though those losses are not recognized in the mitogenome of *D. indicus*. In angiosperms, mitochondrial gene losses have occurred mainly in ribosomal protein (*rpl* or *rps*) and succinate dehydrogenase (*sdh*) genes [17]. Examples of the loss of other kinds of genes are very scarce in angiosperms. The loss of *cox2* occurred in a papilionoid clade in Fabaceae [61]. Losses of some *atp* and *nad* genes were reported from several plant lineages, but not in autotrophic angiosperms [19]. Thus, the missing *atp9* and *nad9* genes in Rubiaceae are notable and need further verification with complete mitogenomes.

Rubiaceae species may have experienced lineage-specific losses of multiple mitochondrial genes over evolutionary times. In this study, however, we focused on a gene loss pattern of *rps7* in the Rubiaceae, since Rydin et al. (2017) [21] did not produce contigs for six genes (*rpl2*, *rpl10*, *rpl16*, *rps2*, *rps11*, *rps14*, *rps19* and *sdh3*), which were likely deleted or pseudogenized during diversification in the family. Our analysis reveals that the loss of *rps7* occurred at least five times in the family (Figure 3). Four of these appear to be recent, when considering they are from four phylogenetically independent genera (*Rondeletia*, *Posoqueria*, *Retiniphyllum* and *Jackiopsis*). The other event occurred in the common ancestor of 21 taxa and the timing was likely before the Paleogene (66–55 million years ago) based on the divergence time estimation of Rydin et al. (2017) [21]. Nonetheless, seven taxa (including *D. indicus*) still retain pseudogenized (but recognizable) copies of *rps7* (Figure 4). These copies shared a single nucleotide insertion, causing a reading frameshift from the early 5′ site in the gene, suggesting that this mutation may be accountable for the pseudogenization early in this clade.

A *trans* configuration of nad1i728 is detected from *D. indicus* (Figure 5). There are two kinds of *trans* configurations of the intron: the so-called wheat type (breakpoint = between *nad1* exon 4 and *matR*) [62] and petunia type (breakpoint = between *matR* and *nad1* exon 5) [63]. The configuration of nad1i728 of *D. indicus* matches the petunia type, observed from several clades of eudicots [34]. Our analysis with an expanded taxon sampling in the Rubiaceae (Table 4) shows taxa from subfamily Rubioideae also share the petunia type *trans* configuration, while the taxa from the other two subfamilies do not. The previous analysis of Qiu and Palmer (2004) [34] analyzed three genera (*Coffea*, *Ixora* and *Galium*) of Rubiaceae and did not show a *trans* configuration of nad1i728. However, *Galium porrigens* var. *tenue*, tested in this study, showed the *trans* configuration. This might be due to the fact that the genus *Galium* is not monophyletic [64]. Further studies are required to understand the phylogenetic distribution of the *trans* configuration of nad1i728 in Rubiaceae.

Two distinct mitogenome structures are revealed in *D. indicus* (Table 5, Figure 6a). Here, we presented a plausible scenario based on the two-step recombination of direct repeats (Figure 6b) with reference to the mechanism proposed for episome relocation [31]. The frequency of recombination of repeats is positively correlated with the repeat unit size [65]. Thus, the alternative subgenome configuration, mediated by large direct repeats (e.g., DR1 pair: 3500 bp ×2), is considered reversible in a single individual [66]. Accordingly, the recombination of short direct repeats (DR2 pair: 61 bp ×2), considered non-reversible [40], is critical in forming an alternative mitogenome structure B-type in *D. indicus*. Once the alternative structure is generated, it can be maintained as a minor type or discarded, but there is also a chance of becoming a major type between generations [31,32].

We detected the co-existence of two types (as major and minor types) within each of three individuals of *D. indicus*. This structural heteroplasmy suggests that the different genome structures may represent a change of frequencies, or that the alternative type (i.e., B-type) is independently generated multiple times. In either case, the structural differences in the genome are unlikely to reflect phylogenetic relationships. The short mutational steps in TCS analysis (Figure 7), leading to DIM-Kyu2 (major structure: B-type) (Table 5) from the other individual in cluster2, also support our argument.

On the other hand, the TCS analysis shows a substantial divergence of two clusters essentially corresponding to two morphological groups (var. *indicus* versus var. *microphyllus*), except for DII-Je1. This exception opens a question about the identity of the *D. indicus* individuals in Jeju Island. One of the plausible explanations for this phenomenon is that the DII-Je1 with the “var. *indicus*-like” morphology may have formed through convergent evolution to adapt to the local environment of Jeju Island. Several factors can affect their morphology. The herbivory was pointed out as a major constraint of leaf and spine growth in *D. indicus* [67]. There is a different herbivore composition between Jeju Island and Kyushu [68,69]. The possible connection between change of ploidy level and phenotype [12] cannot also be ruled out. Alternatively, our data may represent the evolutionary history of the mitochondrion only but not a whole organism.

The answer to the question about infraspecific classification in *D. indicus* remains elusive, due to the small number of analyzed samples and the limitation of mitogenome data. A future population-level study that includes genetic markers from three genomic compartments (nucleus, mitochondrion and plastid) will shed light on the complex evolutionary history of the species. The polymorphic sites in complete mitogenomes revealed in this study will be helpful in future studies.

## 4. Materials and Methods

### 4.1. Sampling and Sequencing

Six samples of *D. indicus* were collected from natural habitats in Jeju Island, Korea and Kyushu, Japan. *D. indicus* var. *indicus* were collected from Seogwipo-si (33°15′21.15″ N, 126°21′18.58″ E), Jeju, Korea (DII-je1 and DII-je2) and Kuma-gun (32°15′40.89″ N, 130°46′58.53″ E), Kumamoto Prefecture, Japan (DII-kyu1 and DII-Khu2), respectively. *D. indicus* var. *microphyllus* was collected from Nobeoka-shi (32°41′10.24″ N, 131°47′14.42″ E), Miyazaki Prefecture, Japan (DIM-Kyu1 and DIM-Kyu2). The voucher specimens (Voucher no. LeeDI-191211~191216) were stored in the herbarium at the department of Biology Education, Chonnam National University. Total genomic DNA was extracted from the dried leaf of each sample with a DNeasy Plant Mini Kit (Qiagen, Seoul, Korea). Genomic libraries were constructed and sequenced using the Illumina Miseq platform (LAS, Seoul, Korea) and MGI-seq 2000 platform (LAS, Seoul, Korea) following each platform’s protocols.

### 4.2. Mitogenome Assembly and Annotation

We largely followed the method for mitogenome assembly described in Choi et al. (2019) [20]. Plastome-filtered reads were generated for each individual by read mapping to the recently published plastome sequence of *D. indicus* (MW548283.1) [70] in Geneious Prime 2021.0.3 (https://www.geneious.com/; accessed on 30 April 2021). *De novo* genome assemblies were conducted for each individual based on plastome-filtered reads. Gaps between mitochondrial contigs were filled by polymerase chain reaction (PCR) and Sanger sequencing. Primers (Table S1) for PCR were designed using Primer3 2.3.7 [71]. Annotation of rRNAs, PCGs and introns was conducted based on a mitogenome of *Liriodendron tulipifera* (NC_021152) in Geseq [72]. The annotation for tRNAs was also checked with tRNAscan-SE v2.0 [73]. Plastid genes in mitogenomes were annotated based on the plastome of *D. indicus* (MW548283.1) in Geseq. Repeat sequences (minimum length = 30 bp; maximum mismatches = 10 %) were estimated in Geneious Prime. The amount of MIPTs was assessed by BLASTN [74] searches using a plastome of *D. indicus* (MW548283.1) as a query with a word size of 7 and an e-value of 1e-^6^. BLAST hits with sequence identity higher than 90% were retained.

### 4.3. Gene and Intron Content Comparison with Representative Angiosperms

Nine previously published mitogenomes of angiosperms were acquired to compare the gene content of *D. indicus* mitogenomes with the related taxa. Except for *Liriodendron tulipifera* (NC_021152.1), the other eight species were the same, analyzed in Park et al. (2014) [75], and we followed their determination on the mitochondrial genes of the species. The gene status of 41 mitochondrial genes was compared in a total of ten taxa.

### 4.4. Phylogenetic Analysis and rps7 Investigation in Rubiaceae

To infer phylogenetic relationships among the Rubiaceae taxa based on mitochondrial sequences, concatenated dataset of 38 regions from each of 60 taxa (including outgroup of *Mostuea* sp. and *Asclepias syriaca* and *Rhazya stricta*) generated from Rydin et al. (2017) [21] were acquired. From one individual of *D. indicus* (DII-Kyu1), 38 mitochondrial regions corresponding to the dataset of Rydin et al. (2017) [21] were collected and concatenated. In total, a concatenated sequence dataset of mitochondrial regions from 61 taxa was aligned by MAFFT v.7.017 [76] using default options. The ambiguously aligned or highly diverged sequences were trimmed using Gblocks 0.91b [77] with default options. Maximum likelihood (ML) analyses were conducted using IQ-TREE v1.6.12 [78] with 1000 bootstrap replications and an appropriate nucleotide substitution model was automatically selected.

The status of *rps7* was plotted on the branch, leading to a taxon or taxa showing the gene loss in the phylogenetic tree generated from IQ-TREE. The pseudogenized copies *rps7* of seven taxa were aligned with an intact copy of *Ophiorrhiza mungos* using MAFFT with default options.

### 4.5. Investigation on the Configuration of nad1i728 in Rubiaceae

The *nad1* exon4-nad1i728 (including *matR*)-*nad1* exon5 region of *Asclepias syriaca* (NC_022796.1) and its corresponding two regions of *D. indicus* (DII-Kyu1) were aligned with MAFFT using default options.

To investigate the phylogenetic distribution of the *trans* configuration of nad1i728 in Rubiaceae, ten publicly available NGS reads from three subfamilies were acquired from the sequence read archive (https://www.ncbi.nlm.nih.gov/sra; accessed on 30 April 2021) (Table 4). Each of the reads was mapped to a reference *nad1* exon4-nad1i728 (including *matR*)-*nad1* exon5 region of *A. syriaca* (NC_022796.1) with medium-low sensitivity in Geneious Prime. Unmapped reads were also kept if one read of a pair of unmapped reads was mapped. The sets of collected reads were assembled with medium–low sensitivity in Geneious Prime. Assembled mitochondrial contigs were compared with mitogenomes of *A. syriaca* and *D. indicus* (DII-Kyu1) and their configuration of nad1i728 was determined.

### 4.6. Mitogenome Sequence and Structural Divergence in Damnacanthus Indicus

The mitogenome structure of six individuals of *D. indicus* was analyzed by Mauve alignment with a progressive algorithm [79]. To detect intra-individual structural heteroplasmy, mediated by recombination of DR2 copies (61 bp ×2), raw reads of six individuals were mapped to DR2 motif with medium–low sensitivity in Geneious Prime. Reads with enough length that covered DR2 motif, as well as its flanking 30 bp up and downstream regions, were subsampled in each set of total mapped reads. The ratios of alternative mitogenome structural types in six individuals were analyzed by comparing each of the subsampled reads to four junctional regions of DR2 motifs in DII-Kyu1 (A-type) and DIM-Kyu2 (B-type).

We manually rearranged local collinear blocks (LCBs) of DIM-Kyu2 (B-type) as A-type to align whole mitogenomes. Then, whole mitogenomes were aligned by MAFFT with default options. Based on this alignment, the TCS network was generated by POPART [80].

## 5. Conclusions

The mitogenome evolution has not been studied well in both higher and lower-level taxonomic ranks in angiosperms. Our study represents the attempt to elucidate mitochondrial gene and intron content evolution in Rubiaceae with complete mitogenomes. Mitogenomes of *D. indicus* lost nine PCGs and one of the losses (i.e., *rps7*) dates back to before the Paleogene in Rubiaceae evolution. Our comparative analysis reveals that a shift from *cis* to *trans* configuration of nadi728 (as the petunia type) marks subfamily Rubioideae in the analyzed Rubiaceae taxa. The phylogenetic distribution of this mutation should be further investigated with a more comprehensive sampling of the mitogenomes in the family. In addition, our evaluation of the mitogenome variation of multiple individuals challenges the current infraspecific classification of *D. indicus,* which is based mainly on vegetative morphologies and provides a substantial number of phylogenetically informative characteristics.

## Figures and Tables

**Table 1:** The sequencing information of six individuals of *Damnacanthus indicus*.

Acronym	Infraspecific Classification	Origin	Sequencing Platform	Number of Raw Reads
DII-Kyu1	var. *indicus*	Kyushu, Japan	DNB (MGI)	49,788,516
DII-Kyu2	var. *indicus*	Kyushu, Japan	Miseq (Illumina)	7,603,662
DII-Je1	var. *indicus*	Jeju, Korea	DNB (MGI)	47,817,598
DII-Je2	var. *indicus*	Jeju, Korea	Miseq (Illumina)	8,196,070
DIM-Kyu1	var. *microphyllus*	Kyushu, Japan	DNB (MGI)	44,651,432
DIM-Kyu2	var. *microphyllus*	Kyushu, Japan	Miseq (Illumina)	7,127,138

**Table 2:** Mitochondrial genome assembly statistics of six individuals of *Damnacanthus indicus*.

Acronym	Length (bp)	Coverage	Repeat (bp)	Plastid Origin (bp)	GenBank Accession
DII-Kyu1	419,435	275	17,822	22,689	MZ285073
DII-Kyu2	419,429	71	17,822	22,688	MZ285074
DII-Je1	417,815	300	17,832	22,705	MZ285071
DII-Je2	419,010	66	18,082	22,487	MZ285072
DIM-Kyu1	417,661	250	17,750	22,706	MZ285075
DIM-Kyu2	417,816	82	17,832	22,706	MZ285076

**Table 3:** The status of 17 variable mitochondrial protein-coding genes of *Damnacanthus indicus* and representatives of angiosperms.

Species	Gene Status ^a^																
	*rpl2*	*rpl5*	*rpl10*	*rpl16*	*rps1*	*rps2*	*rps3*	*rps4*	*rps7*	*rps10*	*rps11*	*rps12*	*rps13*	*rps14*	*rps19*	*sdh3*	*sdh4*
*Damnacanthus indicus*	●	●	-	●	●	-	●	●	●	●	-	●	●	●	-	-	●
*Rhazya stricta*	●	●	●	●	●	-	●	●	●	●	-	●	●	●	●	●	●
*Asclepias syriaca*	●	●	●	●	●	-	●	●	●	●	-	●	●	-	●	●	●
*Nicotiana tabacum*	●	●	●	●	●	-	●	●	●	●	-	●	●	●	-	●	●
*Mimulus guttatus*	●	●	●	●	-	-	●	●	-	●	-	●	●	●	-	●	●
*Boea hygrometrica*	●	●	●	●	-	-	●	●	●	●	-	●	●	●	-	●	●
*Daucus carota*	-	●	●	●	●	-	●	●	●	-	-	●	●	-	-	-	-
*Helianthus annuus*	-	●	●	●	-	-	●	●	-	-	-	●	●	-	●	-	●
*Vaccinium macrocarpon*	●	●	●	●	●	-	●	●	-	●	-	●	●	●	●	●	●
*Liriodendron tulipifera*	●	●	●	●	●	●	●	●	●	●	●	●	●	●	●	●	●

^a^ (● = intact, ● = pseudogenized, - = absent).

**Table 4:** The status of nad1i728 in ten representatives of three subfamilies in Rubiaceae.

Species	Subfamily	Nad1i728 (*cis*/*trans*)	GenBank Accession	SRA ^a^ Run Number	Original Publication of SRA Data
*Galium porrigens* var. *tenue*	Rubioideae	*Trans*	MZ292736 and MZ292737	SRR9961329	Burge (2020) [46]
*Ophiorrhiza* *pumila*	Rubioideae	*Trans*	MZ292738 and MZ292739	DRR194739	Rai et al. (2021) [47]
*Gynochthodes cochinchinensis*	Rubioideae	*Trans*	MZ292740 and MZ292741	SRR12903483	Bautista et al. (2021) [48]
*Foonchewia* *coriacea*	Rubioideae	*Trans*	MZ292742 and MZ292743	SRR12917150	Zhang et al. (2021) [49]
*Mitragyna* *speciosa*	Cinchonoideae	*Cis*	MZ292744	SRR12673030	Brose et al. (2021) [50]
*Chiococca* *alba*	Cinchonoideae	*Cis*	MZ292745	SRR9087163	Lau et al. (2020) [51]
*Corynanthe* *mayumbensis*	Cinchonoideae	*Cis*	MZ292746	SRR8690411	Erickson (2020) [52]
*Catunaregam* *spinosa*	Ixoroideae	*Cis*	MZ292747	SRR7121945	Liu et al. (2019) [53]
*Coffea arabica*	Ixoroideae	*Cis*	MZ292748	SRR7637601	Tran et al. (2018) [54]
*Diplospora* *mollissima*	Ixoroideae	*Cis*	MZ292749	SRR7121910	Liu et al. (2019) [53]

^a^ Sequence read archive (https://www.ncbi.nlm.nih.gov/sra; accessed on 30 April 2021).

**Table 5:** Statistics for the alternative mitogenome structure in six individuals of *Damnacanthus indicus*.

Acronym	Analyzed Reads (Number)	A-Type (Number)	A-Type (%)	B-Type (Number)	B-Type (%)
DII-Kyu1	114	113	99.1	1	0.9
DII-Kyu2	78	77	98.7	1	1.3
DII-Je1	116	116	100.0	0	0.0
DII-Je2	70	70	100.0	0	0.0
DIM-Kyu1	101	101	100.0	0	0.0
DIM-Kyu2	121	6	5.0	115	95.0

## Data Availability

Data available in a publicly accessible repository. GenBank accession numbers for the new sequences are MZ285071-MZ285076 and MZ292736-MZ292749.

## Supplementary Materials

The following are available online at https://www.mdpi.com/article/10.3390/ijms22137237/s1. Table S1: Information for primers and PCR experiments of mitochondrial genome completion.
